# Resistance to CTLA-4 checkpoint inhibition reversed through selective elimination of granulocytic myeloid cells

**DOI:** 10.18632/oncotarget.18437

**Published:** 2017-06-11

**Authors:** Paul E. Clavijo, Ellen C. Moore, Jianhong Chen, Ruth J. Davis, Jay Friedman, Young Kim, Carter Van Waes, Zhong Chen, Clint T. Allen

**Affiliations:** ^1^ Tumor Biology Section, Head and Neck Surgery Branch, National Institute on Deafness and Other Communication Disorders, National Institutes of Health, Bethesda, MD, USA; ^2^ Department of Otolaryngology-Head and Neck Surgery, Johns Hopkins School of Medicine, Baltimore, MD, USA

**Keywords:** MDSCs, CTLA-4, T-cell inflamed, rejection, TCGA

## Abstract

**Purpose:**

Local immunosuppression remains a critical problem that limits clinically meaningful response to checkpoint inhibition in patients with head and neck cancer. Here, we assessed the impact of MDSC elimination on responses to CTLA-4 checkpoint inhibition.

**Experimental Design:**

Murine syngeneic carcinoma immune infiltrates were characterized by flow cytometry. Granulocytic MDSCs (gMDSCs) were depleted and T-lymphocyte antigen-specific responses were measured. Tumor-bearing mice were treated with MDSC depletion and CTLA-4 checkpoint blockade. Immune signatures within the human HNSCC datasets from The Cancer Genome Atlas (TCGA) were analyzed and differentially expressed genes from sorted human peripheral MDSCs were examined.

**Results:**

gMDSCs accumulated with tumor progression and correlated with depletion of effector immune cells. Selective depletion of gMDSC restored tumor and draining lymph node antigen-specific T-lymphocyte responses lost with tumor progression. A subset of T-cell inflamed tumors responded to CTLA-4 mAb alone, but the addition of gMDSC depletion induced CD8 T-lymphocyte-dependent rejection of established tumors in all treated mice that resulted in immunologic memory. MDSCs differentially expressed chemokine receptors. Analysis of the head and neck cancer TCGA cohort revealed high CTLA-4 and MDSC-related chemokine and an MDSC-rich gene expression profile with a T-cell inflamed phenotype in > 60% of patients. CXCR2 and CSF1R expression was validated on sorted peripheral blood MDSCs from HNSCC patients.

**Conclusions:**

MDSCs are a major contributor to local immunosuppression that limits responses to checkpoint inhibition in head and neck cancer. Limitation of MDSC recruitment or function represents a rational strategy to enhance responses to CTLA-4-based checkpoint inhibition in these patients.

## INTRODUCTION

Due to high genomic alteration rates, head and neck squamous cell carcinomas (HNSCCs) are predicted to have a high mutation-derived neoantigen repertoire [1]. Accordingly, significant subsets of patients with carcinogen-associated HNSCC display a T-cell inflamed phenotype [2, 3]. Patients with a T-cell inflamed tumor microenvironment (TME) are more likely to respond to immunotherapy [4], yet only a subset of HNSCC patients respond to checkpoint inhibition [5]. Immunosuppression within the HNSCC TME is well established [6], likely contributes to immune escape of antigenic tumor cells, and may facilitate unresponsiveness to checkpoint inhibition in patients with T-cell inflamed tumors.

Several cellular mediators of immunosuppression within the HNSCC TME have been identified, including myeloid derived suppressor cells (MDSCs) and regulatory T-lymphocytes (Tregs) [7–10]. MDSCs are likely recruited to the TME through chemokine signaling [11, 12], expand locally in response to tumor cell secreted semaphorin 4D [13], and mediate suppression of T-lymphocyte function at least through STAT3-dependent production of arginase [14]. MDSCs can be divided into granulocytic or monocytic subtypes based on cell surface marker expression [15], and the relative abundance of each seems to vary amongst tumor types with both phenotypes expanded in patients with HNSCC [14]. Tregs mediate immunosuppression at least through IL-10 and TGFβ secretion and expression of cell surface cytotoxic T-lymphocyte-associated protein 4 (CTLA-4) [9, 16]. While correlative data suggests both MDSCs and Tregs are functionally relevant in HNSCC, controversy exists over their relative contribution to local immunosuppression with in the TME during both tumor development and progression.

Here, we explored the role of MDSCs during tumor progression in carcinogen-induced syngeneic models of HNSCC, and the ability to manipulate these cells to sensitize tumors to checkpoint inhibition. Using mice bearing T-cell inflamed mouse oral cancer 1 (MOC1) tumors and non-T-cell inflamed MOC2 tumors to model both human HNSCC immune phenotypes [17], we demonstrated loss of T-lymphocyte infiltration and antigen-specific function associated with Ly6G^hi^ cell infiltration during tumor progression in T-cell inflamed tumors. Following validation of the immunosuppressive capacity of these cells, we demonstrated that this loss of T-lymphocyte function with tumor progression could be completely rescued with gMDSC depletion. Therapeutically, depletion of gMDSC sensitized T-cell inflamed tumors to CTLA-4-based checkpoint inhibition. Combination treatment resulted in consistent CD8-dependent rejection of established tumors and formation of immunologic memory. These results were not observed in non-T-cell inflamed tumors. Recruitment of gMDSC into T-cell inflamed tumors correlated with CXCL1/CXCR2 chemokine axis components, suggesting a therapeutic target. Finally, we explored The Cancer Genome Atlas (TCGA) HNSCC dataset and offer evidence to support that MDSC and CTLA-4 blockade represent rational therapeutic strategies in T-cell inflamed HNSCCs.

## RESULTS

### Ly6G^hi^ myeloid cells accumulation inversely correlated with effector immunity

**Figure 1:**
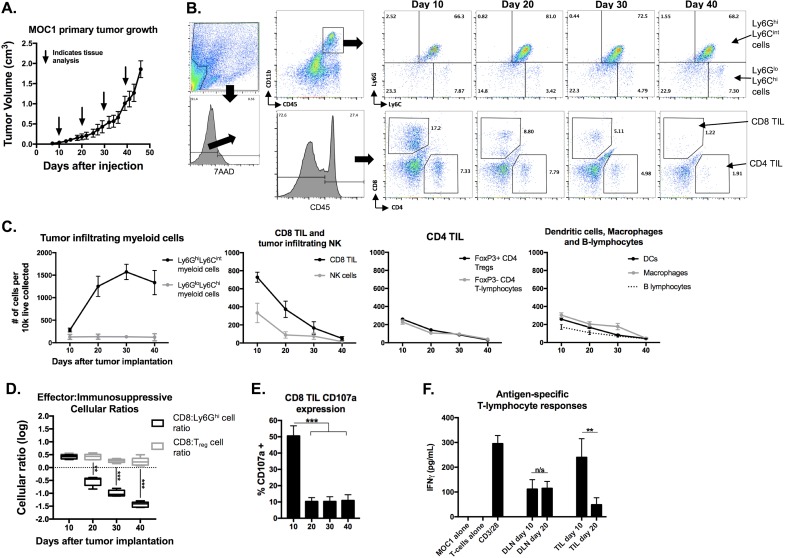
Accumulation of MOC1 tumor Ly6G ^hi^ myeloid cells with tumor progression inversely correlated with accumulation of effector immune cells and T-lymphocyte antigen-specific reactivity MOC1 tumors were harvested at days 10, 20, 30 and 40 (*n* = 5/time point) and analyzed for immune cell infiltration and activation by flow cytometry. **A.**, average MOC1 primary tumor growth curve and tissue harvest time points. **B.**, flow gating strategy and representative dot plots for Ly6G^hi^Ly6C^int^ myeloid cells, Ly6G^lo^Ly6C^hi^ myeloid cells, CD4^+^ and CD8^+^ TIL. **C.**, quantification of myeloid cells, CD8 TIL, NK cells (CD3^−^NK1.1^+^), FoxP3^+/−^ CD4 TIL, DCs (CD11c+CD11b^+/−^PDCA^+/−^), macrophages (CD11b^+^F4/80^+^) and B-lymphocyte (B19^+^B220^+^) infiltration, normalized to number of cells per 1×10^4^ live cells collected. **D.**, box and whiskers plot demonstrating changes in CD8^+^ TIL: Ly6G^hi^ cell ratio and CD8^+^ TIL:Treg (FoxP3^+^CD4^+^ TIL) ratio with tumor progression. **E.**, quantification of CD8^+^ TIL cell surface CD107a positivity by flow cytometry. **F.**, T-lymphocytes were isolated from day 10 and 20 draining lymph nodes and tumors (*n* = 5/group), pooled, and assessed for IFNγ production upon exposure to MOC1 tumor cell antigen; results pooled from two independent assays each with technical triplicates. **. *P* < 0.01; ***, *P* < 0.001. n/s, non-significant.

**Figure 2:**
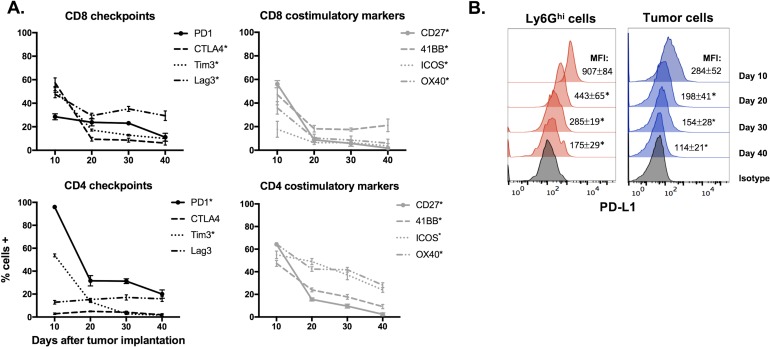
Expression of immune checkpoints and costimulatory markers in the MOC1 tumor microenvironment trended down with tumor progression **A.**, Immune checkpoints (PD1, CTLA-4, Tim3 and Lag3) and costimulatory markers (CD27, 41BB, ICOS and OX40) were measured on CD4^+^ and CD8^+^ TIL from MOC1 tumors at day 10, 20, 30 and 40 after tumor implantation (*n* = 5/time point) *via* flow cytometry. * denotes a statistically significant change (*p* < 0.05) from day 10 to 20. **B.**, representative histograms of PD-L1 expression on MOC1 tumor infiltrating Ly6G^hi^ myeloid and tumor cells with tumor progression. * denotes a statistically significant change (*p* < 0.05) from previous time point.

To assess changes in the tumor microenvironment with tumor progression, we analyzed immune cell infiltration into MOC1 and MOC2 tumors at multiple time points. In T-cell inflamed MOC1 tumors (Figure 1A), increased Ly6G^hi^Ly6C^int^ myeloid (CD11b+) cells but not Ly6G^lo^Ly6C^hi^ myeloid cells correlated with decreased CD8^+^ TIL, Tregs, FoxP3^−^ CD4^+^ TIL, NK cell, dendritic cell, mature macrophage and B-lymphocyte tumor infiltration as tumors progressed (representative dot plots in Figure 1B, quantification in 1C). Representative dot plots of Treg tumor infiltration are shown in Supplementary Figure S1A. Selected flow cytometric findings were validated with immunofluorescence (Supplementary Figure S1B&S1C). Similar findings were found in the spleens of MOC1 tumor bearing mice, with splenomegaly and accumulation of Ly6G^hi^ myeloid cells but not Tregs with tumor progression (Supplementary Figure S2). Within MOC1 tumors, the largest increase in accumulation of Ly6G^hi^ myeloid cells occurred between days 10 and 20 of tumor progression. Evaluated as a ratio with CD8^+^ effector immune cells, CD8:Ly6G^hi^ cell ratios transitioned from positive to negative between days 10 and 20, whereas CD8:T_reg_ ratios stayed positive over time (Figure 1D). CD107a positivity, a measure of TIL degranulation, significantly decreased between days 10 and 20 of MOC1 tumor progression (Figure 1E). While sorted CD8^+^ T-lymphocytes from tumor draining lymph nodes (DLN) maintained similar antigen responsiveness, antigen-specific responses from CD8^+^ TIL were significantly suppressed between days 10 and 20 of tumor progression (Figure 1F). The expression of checkpoints on TIL (Figure 2A), as well as PD-L1 expression on tumor cells and tumor infiltrating myeloid cells (Figure 2B) followed the same pattern of decrease with tumor progression. These correlative data suggested that accumulation of Ly6G^hi^Ly6C^int^ myeloid cells correlates with loss of effector immune cell infiltration and function and that these granulocytic cells represent mediators of local immunosuppression within MOC1 tumors.

**Figure 3:**
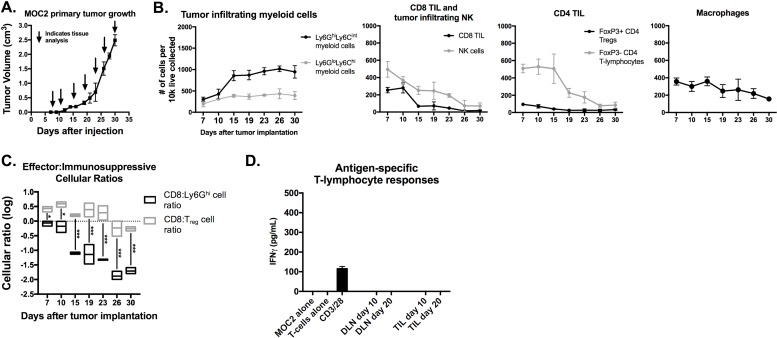
Accumulation of MOC2 tumor Ly6G ^hi^ myeloid cells inversely correlated with accumulation of effector immune cells with tumor progression MOC2 tumors were harvested at days 7, 10, 15, 19, 23, 26 and 30 (*n* = 3/time point) and analyzed for immune cell infiltration by flow cytometry. A, average MOC2 primary tumor growth curve and tissue harvest time points. B, quantification of gMDSC, mMDSC, CD8^+^ TIL, NK cells, FoxP3 positive and negative CD4^+^ TIL and macrophages, normalized to number of cells per 1×10^4^ total live cells collected. **C.**, box and whiskers plot demonstrating changes in CD8^+^ TIL:gMDSC ratio and CD8^+^ TIL:Treg (FoxP3^+^CD4^+^ TIL) ratio with tumor progression. **D.**, T-lymphocytes were isolated from day 10 and 20 draining lymph nodes and tumors (*n* = 5/group), pooled, and assessed for IFNγ production upon exposure to antigen on MOC1 tumor cells. *, *p* < 0.05; ***, *p* < 0.001.

Non-T-cell inflamed MOC2 tumors demonstrated a similar pattern of increased Ly6G^hi^Ly6C^int^ myeloid cell but not Treg accumulation with tumor progression that was associated with loss of effector CD8^+^ and CD4^+^ TIL and NK cell infiltration (Figure 3A-3C). However, day 10 and 20 MOC2 DLN T-lymphocytes and TIL were unresponsive when exposed to MOC2 cells (Figure 3D), suggesting either that MOC2 tumor cells lack antigen or the presence of other MOC2 intrinsic mechanisms of resistance to T-cell recognition.

### Ly6G^hi^ myeloid cells potently suppressed T-lymphocyte proliferation and lytic activity

**Figure 4:**
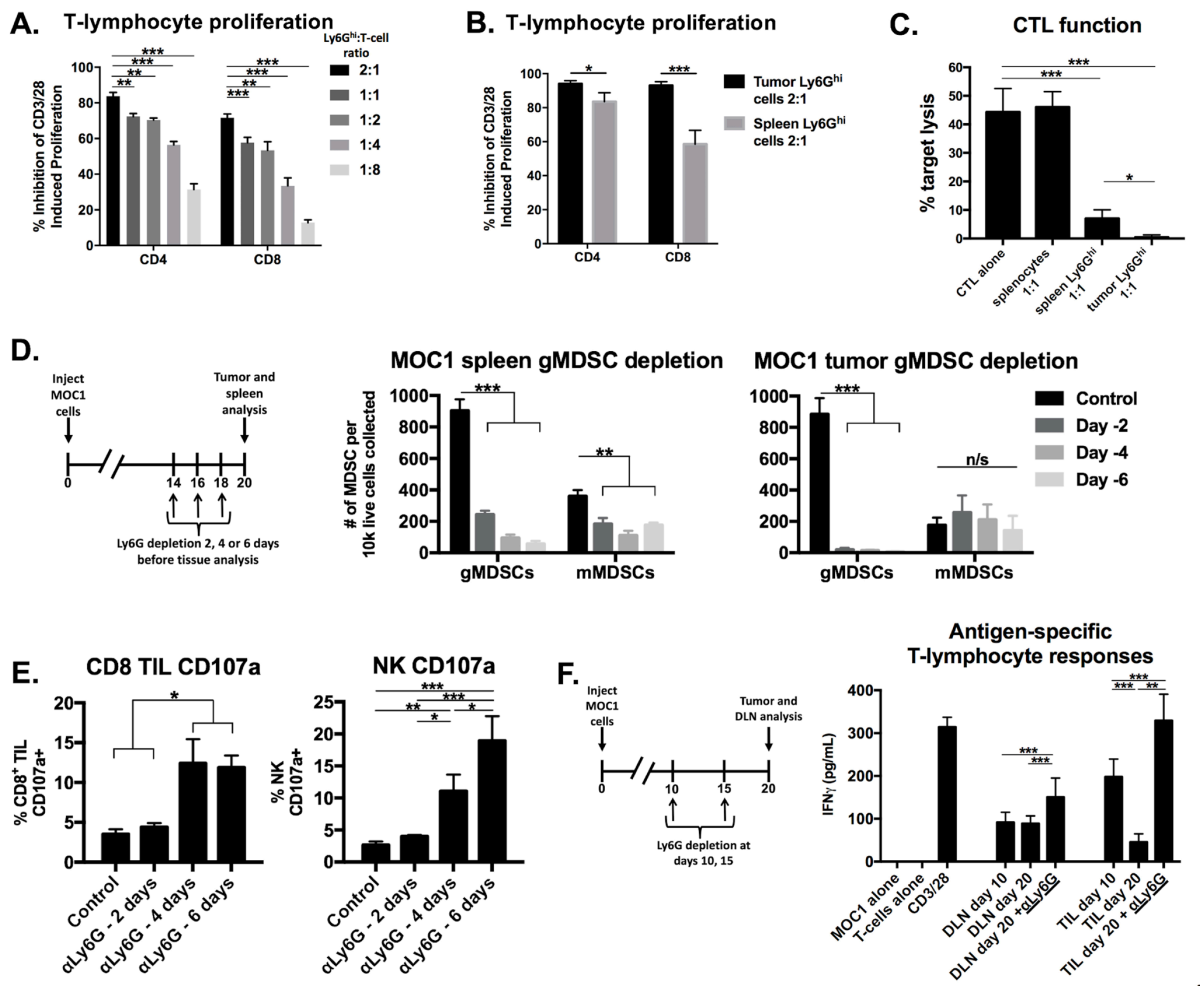
Depletion of immunosuppressive gMDSCs from MOC1 tumor-bearing mice enhanced effector immune cell activation and rescued antigen-specific T-lymphocyte reactivity lost with tumor progression **A.**, isolated splenic Ly6G^hi^ myeloid cells were analyzed for their *ex vivo* ability to suppress CFSE-labelled CD4^+^ and CD8^+^ T-lymphocyte proliferation. Inhibition of proliferation (division index) with different Ly6G^hi^:T-lymphocyte ratios are shown. **B.**, isolated splenic and tumor-infiltrating gMDSCs were assessed for their ability to suppress CD4^+^ and CD8^+^ T-lymphocyte proliferation at a 2:1 Ly6G^hi^ cell:T-lymphocyte ratio. **C.**, isolated splenic and tumor-infiltrating gMDSCs were assessed for their ability to suppress OT-1 CTL killing of SIINFEKL-pulsed EL4 cells. Splenocytes or spleen/tumor Ly6G^hi^ cells were added at a 1:1 ratio to CTLs. **D.**, schematic demonstrating a single injection of Ly6G depleting antibody (clone 1A8, 200 μg/injection) *in vivo* at either day 14, 16 or 18 (6, 4 or 2 days before tissue analysis, respectively) before tissue analysis on day 20. Right bar graphs demonstrate absolute numbers of splenic and tumor MDSC after Ly6G mAb administration. **E.**, CD8^+^ TIL and tumor infiltrating NK cell degranulation (CD107a positivity) was assessed by flow cytometry following gMDSC depletion. F, schematic demonstrating *in vivo* Ly6G depletion at days 10 and 15 with tissue analysis at day 20. Draining lymph node T-lymphocytes and TIL were isolated from mice treated with Ly6G depleting antibody or isotype control, pooled, and assessed for IFNγ production upon exposure to MOC1 tumor cell antigen. All *in vitro* data shown pooled from at least two independent experiments performed in technical triplicate. *, *p* < 0.05; **, *p* < 0.01; ***, *p* < 0.001. n/s, non-significant.

To evaluate if Ly6G^hi^ myeloid cells that accumulated in the periphery and tumors of MOC1 tumor-bearing mice were immunosuppressive, we performed *ex vivo* T-lymphocyte functional assays in the presence of sorted Ly6G^hi^ myeloid cells. The purity and phenotype of these sorted gMDSC have been described [18]. Splenic Ly6G^hi^ cells from MOC1 tumor-bearing mice suppressed CD3/28 stimulated CD4^+^ and CD8^+^ T-lymphocyte proliferation in a dose-dependent fashion (Figure 4A). When evaluated head-to-head at a fixed Ly6G^hi^ to T-lymphocyte ratio, tumor infiltrating Ly6G^hi^ cells suppressed T-lymphocyte proliferation to a significantly greater degree than splenic Ly6G^hi^ cells (Figure 4B). We next assessed the ability of MOC1 sorted Ly6G^hi^ cells to suppress antigen-specific CTL cytolytic capacity, and found that the presence of Ly6G^hi^ cells but not naïve splenocytes significantly inhibited target cell killing by effector CTLs (Figure 4C). Tumor Ly6G^hi^ cells suppressed CTL function to a greater degree than splenic Ly6G^hi^ cells. These data functionally validated Ly6G^hi^ cells in MOC1 tumors as granulocytic myeloid derived suppressor cells (gMDSCs).

### gMDSC depletion rescued loss of T-lymphocyte antigen-specific responses

**Figure 5:**
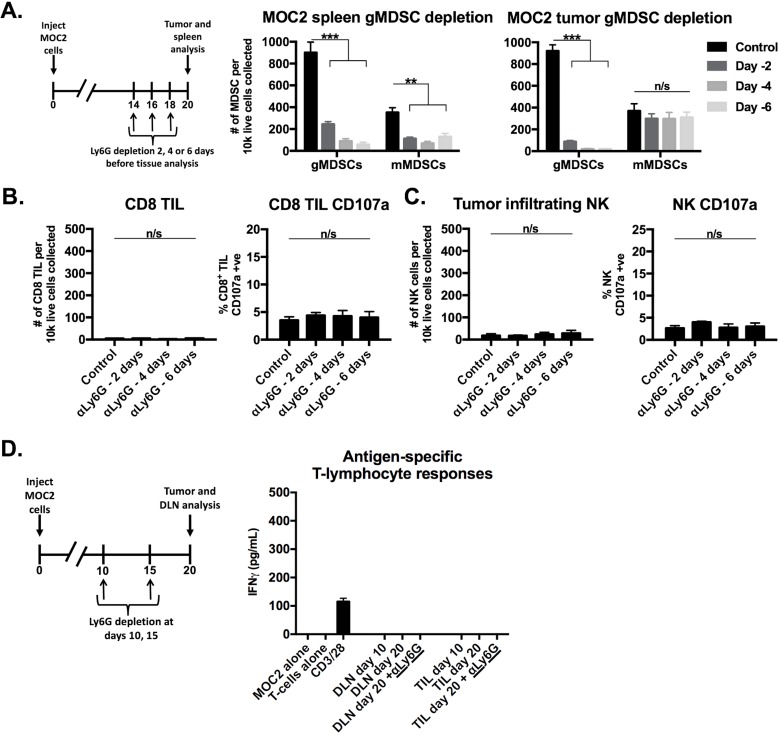
Depletion of gMDSCs from MOC2 tumor-bearing mice did not enhance effector immune cell activation **A.**, schematic demonstrating a single injection of Ly6G depleting antibody (clone 1A8, 200 μg/injection) at either day 14, 16 or 18 (6, 4 or 2 days before tissue analysis, respectively) before tissue analysis on day 20. Right bar graphs demonstrate absolute numbers of splenic and tumor MDSC after Ly6G mAb administration. **B.**, CD8^+^ TIL infiltration and degranulation (CD107a positivity) were assessed by flow cytometry following gMDSC depletion. **C.**, NK cell tumor infiltration and degranulation (CD107a positivity) were assessed by flow cytometry. **D.**, schematic demonstrating Ly6G depletion at days 10 and 15 with tissue analysis at day 20. Draining lymph node T-lymphocytes and TIL were isolated from mice treated with Ly6G depleting antibody or isotype control, pooled, and assessed for IFNγ production upon exposure to MOC2 tumor cells. **, *p* < 0.01; ***, *p* < 0.001. n/s, non-significant.

We next assessed the functional impact of eliminating gMDSC from the MOC1 tumor microenvironment. We validated that antibody clone 1A8 but not clone RB6-8C5 leads to efficient and specific depletion of Ly6G^hi^ myeloid cells but not CD4^+^ or CD8^+^ T-lymphocytes (Supplementary Figure S3). gMDSCs were depleted from both the spleen and to a greater degree from the tumor microenvironment in MOC1 tumor-bearing mice up to 6 days after a single injection of Ly6G antibody (Figure 4D). Following gMDSC depletion in MOC1 tumor-bearing mice, accumulation of CD8^+^ T-lymphocytes and NK cells did not change but demonstrated significantly increased expression of CD107a (Figure 4E). This suggested that eliminating gMDSCs did not enhance accumulation of effector immune cells but rather rescued function. To validate this finding, we sorted T-lymphocytes from MOC1 DLN and TIL with or without gMDSC depletion. The loss of antigen-specific TIL responses observed with tumor progression between days 10 and 20 were completely recovered and enhanced beyond day 10 levels following gMDSC depletion (Figure 4F). DLN T-lymphocyte antigen-specific responses were more modestly enhanced with gMDSC depletion. Conversely, despite similar treatment, depletion of gMDSC from the tumor microenvironment in MOC2 tumor-bearing mice did not enhance CD8^+^ TIL or NK cell CD107a expression or induce antigen specific responses in TIL or DLN T-lymphocytes (Figure 5A-5D). Cumulatively, these data indicated that manipulation of gMDSC within the T-cell inflamed MOC1 tumor microenvironment rescued loss of T-lymphocyte function associated with tumor progression, but had little effect on non-T-cell inflamed MOC2 tumors.

### gMDSCs depletion enhanced tumor rejection following CTLA-4 checkpoint inhibition

**Figure 6:**
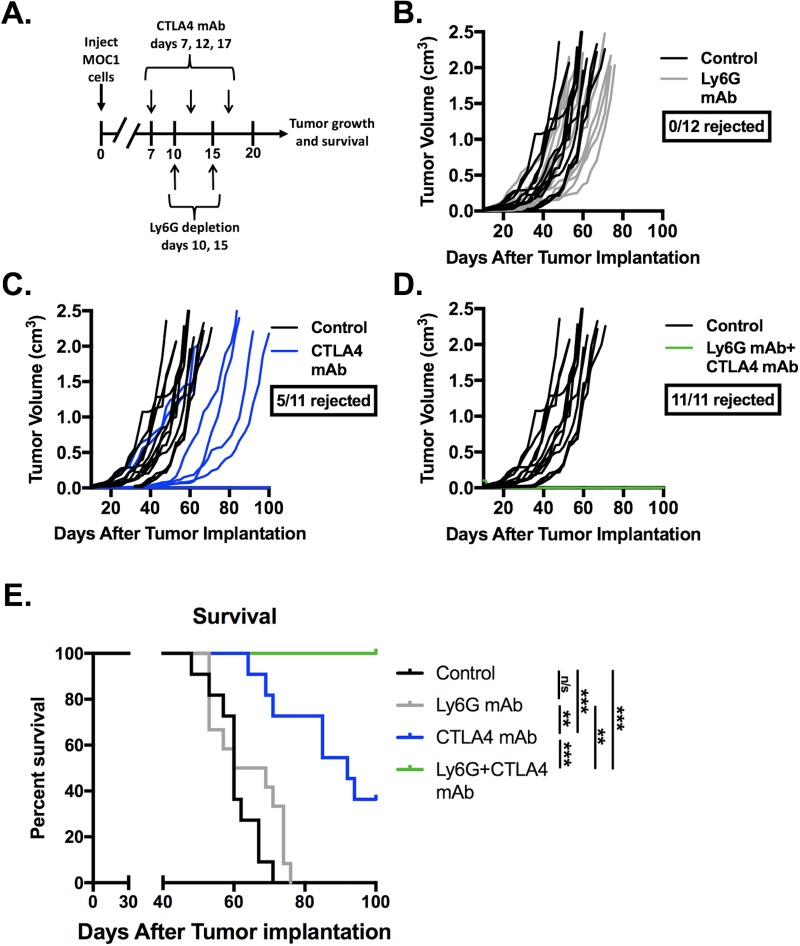
Depletion of gMDSC sensitized MOC1 tumors to CTLA-4 mAb induced tumor rejection Established MOC1 tumors were treated with Ly6G depleting antibody (clone 1A8, 200 μg/injection) and CTLA-4 mAb (clone 9H10, 100 μg/injection), alone or in combination. **A.**, schematic of Ly6G depletion and checkpoint blockade. Primary tumor growth plots demonstrate growth curves for treated MOC1 tumors (colored lines) compared to control (black lines) for Ly6G mAb alone **B.**, or CTLA-4 with **C.** or without **D.** Ly6G mAb. **E.**, survival analysis of treated MOC1 tumor-bearing mice, with statistical significance between treatment groups as indicated. Results pooled results from two independent experiments are shown. **, *p* < 0.01; ***, *p* < 0.001. n/s, non-significant.

**Figure 7:**
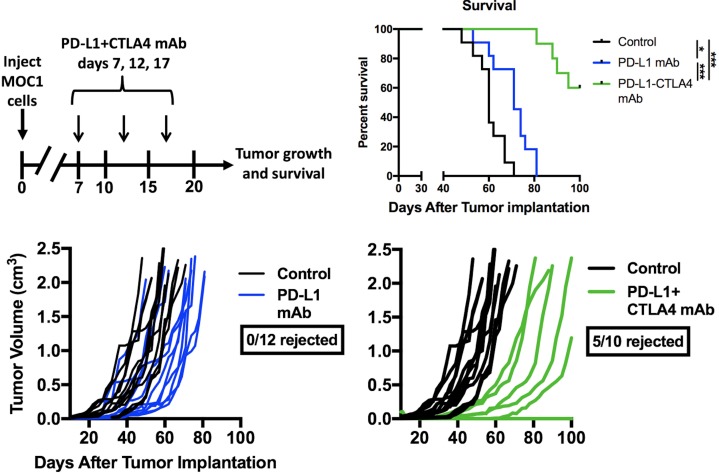
Addition of PD-L1 mAb to CTLA-4 mAb did not enhance MOC1 tumor control or rejection rates Mice bearing established MOC1 tumors were treated with PD-L1 mAb (clone 10F.9G2, 200 μg/injection) alone or in combination with CTLA-4 mAb (clone 9H10, 100 μg/injection) and followed for tumor growth and survival. Treatment schema, individual tumor growth curves and survival are shown. *, *p* < 0.05; ***, *p* < 0.001.

**Figure 8:**
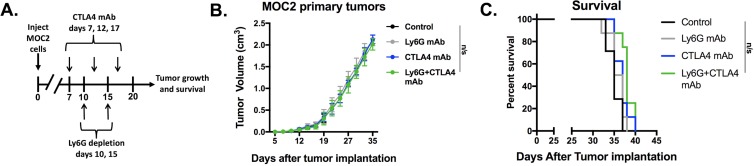
Depletion of gMDSC did not enhance responses to CTLA-4 mAb in MOC2 tumor-bearing mice Established MOC2 tumors were treated with Ly6G depleting antibody (clone 1A8, 200 μg/injection) and CTL-A4 mAb (clone 9H10, 100 μg/injection), alone or in combination. Treatment schema, average tumor growth curves for each treatment condition and survival are shown. n/s, non-significant.

Given evidence that eliminating gMDSC from the tumor environment enhanced T-lymphocyte responsiveness, we first assessed MOC1 primary growth following gMDSC depletion (Figure 6A). Ly6G mAb treatment alone induced little primary tumor growth delay suggesting that other factors within the tumor microenvironment also limited effective anti-tumor immunity (Figure 6B). We next combined gMDSC depletion with CTLA-4 mAb checkpoint inhibition in MOC1 tumor-bearing mice. Treatment with CTLA-4 mAb alone induced tumor rejection in 5 of 11 mice treated (Figure 6C). The addition of gMDSC depletion to CTLA-4 blockade resulted in tumor rejection of all treated MOC1 tumor-bearing mice (Figure 6D), resulting in significantly prolonged survival (Figure 6E). Given that gMDSC express very high levels of PD-L1, we assessed whether enhanced tumor rejection following gMDSC depletion plus CTLA-4 mAb was primarily through elimination of PD-L1 from the TME. Treatment of MOC1 tumor-bearing mice with PD-L1 mAb alone had modest effects (Figure 7). When added to CTLA-4 mAb, PD-L1 mAb did not enhance the rate of tumor rejection over that observed with CTLA-4 mAb alone (50% *vs*. 45%, respectively), suggesting that the enhanced MOC1 tumor elimination observed with gMDSC depletion and CTLA-4 mAb was not fully attributable to elimination of PD-L1 expressed on gMDSC. To reinforce this concept of mechanistic overlap and for biologic comparison, treatment of MOC1 tumor-bearing mice with combination gMDSC depletion and PD-L1 mAb alone produced no rejections and modestly delayed primary tumor growth (Supplementary Figure S4). Combination gMDSC depletion with CTLA-4 mAb checkpoint inhibition produced no delay in primary tumor growth or extension of survival in MOC2 tumor-bearing mice (Figure 8).

**Figure 9:**
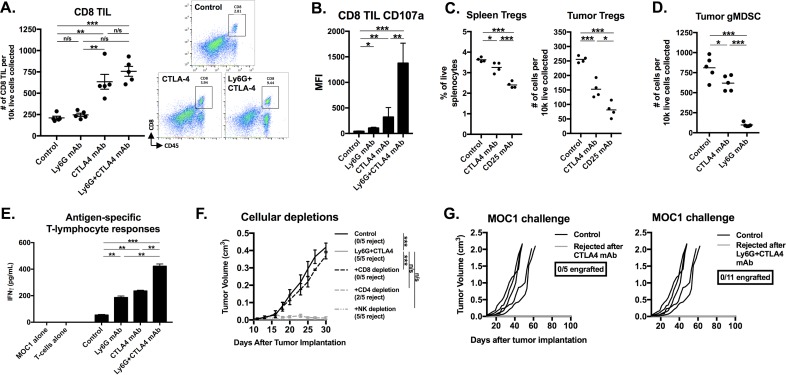
Immune correlative and functional analysis revealed partial Treg depletion, CD8 ^+^ T-lymphocyte dependent tumor rejection, and induction of immunologic memory in MOC1 tumor-bearing mice treated with gMDSC depletion and CTLA-4 mAb **A.**, infiltration of CD8+ TIL following treatment with CTLA-4 mAb with or without Ly6G depletion was quantified (left panel) *via* flow cytometry with representative dot plots on the right. **B.**, CD8^+^ TIL cell surface expression of CD107a was quantified. **C.**, splenic or tumor-infiltrating FoxP3^+^CD4^+^ Tregs were quantified 48 hours after a single injection (200 μg) of either CTLA-4, CD25 or isotype control mAb into mice bearing 7 day-old tumors. **D.**, tumor-infiltrating Ly6G^hi^ gMDSC were quantified 48 hours after a single injection (200 μg) of either CTLA-4, 1A8 or isotype control mAb into mice bearing 20 day-old tumors. **E.**, draining lymph node T-lymphocytes were isolated from treated mice (*n* = 5/condition), pooled, and assessed for IFNγ production upon exposure to MOC1 tumor antigen. **F.**, in separate experiments, tumor-bearing mice with established MOC1 tumors were treated with combination Ly6G and CTLA-4 mAbs with or without antibodies to deplete CD8 (clone YTS169.4, 200 μg/injection, twice weekly), CD4 (clone GK1.5, 200 μg/injection, twice weekly) or NK cells (clone PK136, 200 μg/injection, twice weekly). **G.**, mice that rejected MOC1 tumors after CTLA-4 mAb alone or in combination with Ly6G mAb were challenged with 5×10^6^ parental MOC1 cells (55 days after original MOC1 implantation, approximately 35 days after MOC1 tumor rejection) and followed for tumor engraftment. *, *p* < 0.05; **, *p* < 0.01; ***, *p* < 0.001. n/s, non-significant.

Immune correlative analysis of treated MOC1 tumors revealed increased infiltration of CD8^+^ TIL in groups treated with CTLA-4 mAb alone or in combination with gMDSC depletion (Figure 9A). Cell surface CD107a staining was significantly increased on CD8^+^ TIL within tumors treated with CTLA-4 mAb plus gMDSC depletion (Figure 9B). Given that certain clones of CTLA-4 mAb can deplete CTLA-4 positive Tregs [19, 20], we measured Treg tumor infiltration following CTLA-4 blockade and found significant but incomplete tumor infiltrating Treg depletion compared to that achieved with CD25 mAb treatment (Figure 9C). Peripheral Treg levels were affected to a lesser degree. Tumor infiltrating gMDSC were modestly altered following CTLA-4 treatment compared to specific Ly6G depletion (Figure 9D). T-lymphocytes sorted from DLN in mice treated with CTLA-4 mAb plus gMDSC depletion demonstrated significantly greater antigen-specific responses compared to other treatment cohorts when exposed to MOC1 cellular antigen (Figure 9E). Treatment of MOC1 tumor-bearing mice with CTLA-4 mAb plus gMDSC depletion in the presence of cellular depleting mAbs revealed that tumor rejection is dependent upon CD8^+^ but not CD4^+^ or NK cells (Figure 9F). Further, mice that rejected MOC1 tumors following treatment with CTLA-4 mAb alone or in combination with gMDSC depletion resisted engraftment when challenged with MOC1 tumor cells (Figure 9G). Taken together, these data indicated that gMDSC depletion enhanced CTLA-4 mAb induced CD8^+^ T-lymphocyte tumor infiltration and activation, DLN T-lymphocyte antigen-specific activation, and CD8-dependent tumor rejection with formation of immunologic memory in T-cell inflamed MOC1 but not non-T-cell inflamed MOC2 tumors.

### gMDSCs appeared to be recruited into tumors through the CXCR2 chemokine axis

**Figure 10:**
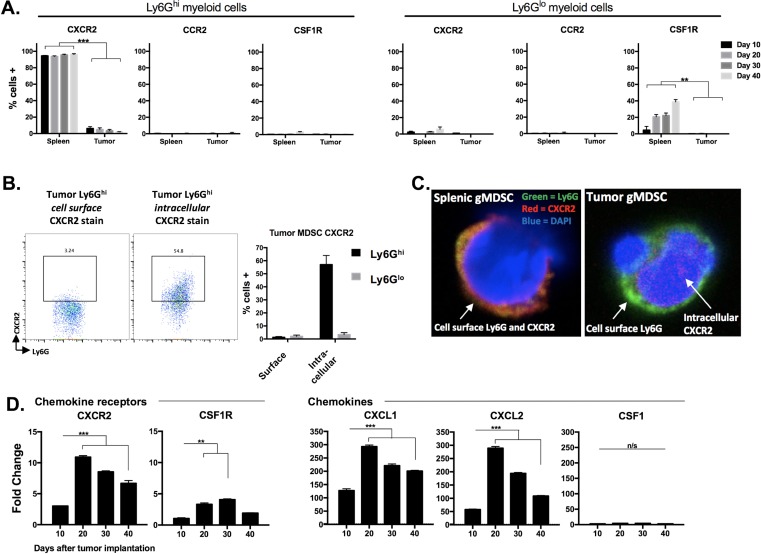
gMDSC appear to be recruited into the tumor microenvironment through CXCR2 signaling **A.**, spleens and tumors from MOC1 tumor-bearing mice were harvested at day 10, 20, 30 and 40 and MDSCs were analyzed for cell surface CXCR2, CCR2 and CSF1R expression (*n* = 5/time point). **B.**, representative dotplots of isolated tumor gMDSCs subjected to cell surface or intracellular (after fixation and permeabilization) CXCR2 staining. Quantification (bar graph) is shown below. Right photomicrographs (63x) demonstrate Ly6G (green) and CXCR2 (red) staining on isolated splenic gMDSC without fixation and permeabilization (top) and on isolated tumor gMDSC with fixation and permeabilization (bottom). **C.**, unsorted MOC1 tumor tissues were collected at days 10, 20, 30 and 40, RNA was isolated from digested single cell suspensions, and qRT-PCR analysis was used to measure chemokine receptor (CXCR2, CSF1R, referenced to day 10 CSF1R levels) and ligand (CXCL1, CXCL2, CSF1, referenced to day 10 CSF1 levels) transcript levels (*n* = 3/time point). **, *p* < 0.01; ***, *p* < 0.001.

To evaluate possible therapeutic targets to block MDSC recruitment into the tumor microenvironment, we determined cell surface expression of common myeloid chemokine receptors on splenic and tumor Ly6G^hi^ and Ly6G^lo^ myeloid cells in MOC1 tumor-bearing mice. A subset of splenic Ly6G^lo^ myeloid cells expressed CSF1R, but nearly all splenic Ly6G^hi^ myeloid cells expressed CXCR2 (Figure 10A, 10B). CXCR2 was not present on the surface of Ly6G^hi^ myeloid cells within the tumor microenvironment. Based upon previous work demonstrating chemokine receptor internalization following ligation [21], we explored localization of CXCR2 and found CXCR2 to be internalized in Ly6G^hi^ myeloid cells that had trafficked into MOC1 tumors (Figure 10C). We next measured whole tumor RNA levels of the chemokine receptors CXCR2 and CSF1R as well as the cognate chemokines for these receptors, CXCL1/CXCL2 and CSF1, respectively. Consistent with patterns of heavy Ly6G^hi^ gMDSC infiltration, MOC1 tumors expressed increased transcript levels of CXCR2 and the CXCR2 ligands CXCL1 and CXCL2, with a significant increase in expression between days 10 and 20 of tumor progression (Figure 10D). Cumulatively, and combined with previous reports from our laboratory [22], these correlative data suggest that the CXCL1/CXCR2 chemokine signaling axis may be a principal driver of gMDSC recruitment into the MOC tumor microenvironment.

### Subsets of human HNSCCs demonstrate an MDSC-rich gene expression profile

**Figure 11:**
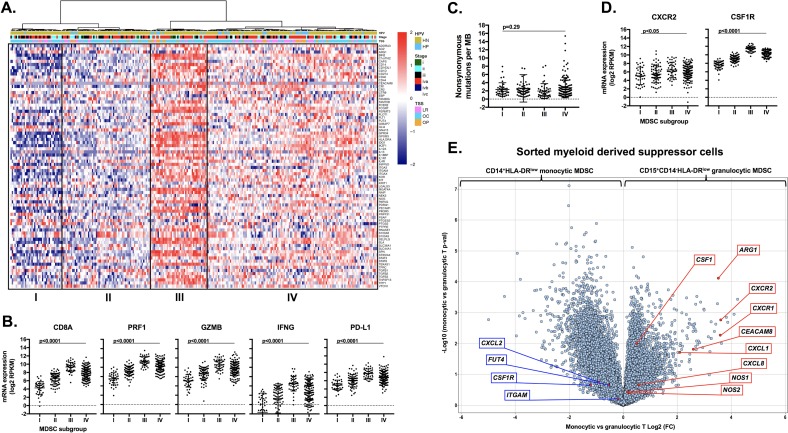
Analysis of human HNSCC data revealed high CXCR2 axis and checkpoint expression and identified MDSC rich subgroups that are T-cell inflamed Immune signatures of 297 HNSCC patient samples by RNAseq from the TCGA cohort were analyzed. **A.**, expression profiles of 77 MDSC-associated genes are presented by heatmap (y-axis) [39] where red indicates relative gene overexpression and blue indicates relative gene underexpression compared to means for each gene. Supervised hierarchical clustering revealed four human MDSC subgroups. Tumor site (NM, normal mucosa; OC, oral cavity; LR, larynx; OP, oropharynx) and HPV status (HP, HPV positive; HN, HPV negative) are indicated at the top of the heatmap. **B.**, RNA expression patterns of effector CD8^+^ T-lymphocyte associated genes (*CD8a, Prf1, Gzmb, Ifng, CD274/PD-L1*) based on MDSC gene expression clustering subgroup, analyzed for significance by 1-way ANOVA. **C.**, distribution of mutational burden by MDSC gene expression clustering subgroup. **D.**, RNA expression patterns of chemokine receptors (*CXCR2 and CFS1R*) based on MDSC gene expression clustering subgroup. **E.**, volcano plot displaying the magnitude (x-axis, fold change) and significance (y-axis, p-value) of differential gene expression between sorted peripheral blood monocytic and granulocytic MDSCs from four patients with advanced-stage pharyngeal SCC.

**Figure 12:**
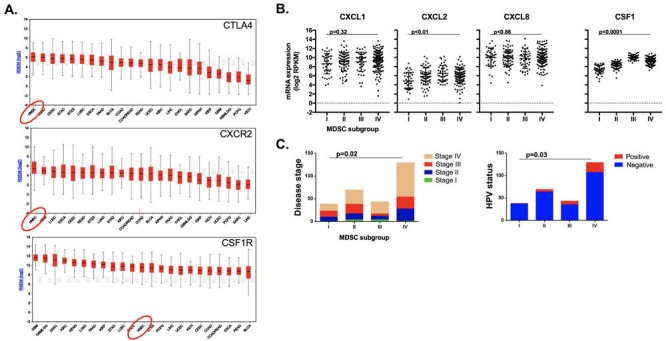
MDSC rich subgroups correlated with CXCR2 axis components, disease stage and HPV status **A.**, average gene expression profiles *CTLA4*, *CXCR2* and *CSF1R* genes amongst 23 tumor types were ranked and plotted based upon their RSEM (log2) value. **B.**, RNA expression patterns of the chemokines *CXCL1*, *CXCL2*, *CXCL8* and *CSF1* based on MDSC gene expression clustering subgroup, analyzed for significance by 1-way ANOVA. **C.**, distribution of clinical parameters by MDSC gene expression clustering subgroup, analyzed for significance by χ-square analysis.

To explore the translational potential of targeting MDSCs as an approach to sensitize human HNSCC to checkpoint inhibition, we analyzed TCGA RNASeq data. Supervised clustering of HNSCC patients based upon expression of a validated MDSC gene signature resulted in the identification of 4 subgroups (Figure 11A). Subgroups I and II represented tumors with a relatively low MDSC gene expression signature, whereas subgroups III and IV demonstrated a high MDSC gene expression profile. A high MDSC gene signature (groups III and IV) correlated with high *CD8a*, *Prf1*, *Gzmb*, *Ifng* and *CD274* (PD-L1) gene expression indicative of an underlying CD8 T-lymphocyte response within HNSCC tumors (Figure 11B). Mutational burden did not differ between MDSC subgroups (Figure 11C). MDSC high subgroups had higher expression of the chemokine receptors CXCR2 and CSF1R (Figure 11D). To validate our murine chemokine expression findings, we performed expression analysis on gMDSC and mMDSC sorted from the peripheral blood of patients with advanced pharyngeal SCC (Figure 11E). Sorted gMDSC expressed high levels of *CXCR2* and *ARG1*, whereas *CSF1R* was expressed to a greater degree in mMDSC. Expression of CTLA-4 and CXCR2 was the highest and among the highest for CSF1R expression within the TCGA HNSCC cohort compared to other tumor types (Figure 12A). Expression of chemokines CXCL2 and CSF1 within HNSCC tumors was highest in the MDSC-rich subgroups. Analysis of clinical parameters revealed that tumors displaying a high MDSC gene expression profile were more likely to be HPV positive and from patients with advanced stage (III/IV) disease (Figure 12B & 12C). Collectively, human HNSCCs with the most T-cell inflamed tumors clustered to MDSC subgroups III and IV with the highest MDSC gene signature, suggesting that targeting MDSCs to sensitize these T-cell inflamed tumors to immune activating treatments such as checkpoint inhibition may represent a valid therapeutic strategy.

## DISCUSSION

Objective responses to checkpoint inhibition tend to be durable, but only a small subset of patients respond [5]. Enhancing the percentage of patients that demonstrate durable tumor control would likely have a significant impact on HNSCC disease-specific morbidity and mortality. Patients with T-cell inflamed tumors are more likely to respond to checkpoint inhibitors [4, 23], but a better understanding why many patients with T-cell inflamed tumors and most patients with non-T-cell inflamed tumors don't respond is critical [24]. Here, *via* specific depletion of Ly6G^hi^ cells, we demonstrated that gMDSCs are a major driver of T-lymphocyte suppression within the TME of T-cell inflamed MOC1 tumors. We additionally demonstrated that responses to CTLA-4-based checkpoint inhibition are significantly enhanced when gMDSCs are eliminated. Mechanistically, gMDSC directly suppressed T-lymphocyte proliferation and cytolytic capacity in *ex vivo* functional assays. Importantly, the effects of gMDSC depletion were not solely dependent upon PD-L1 depletion despite robust PD-L1 expression on gMDSC, as the addition of PD-L1 to CTLA-4 mAb did not produce the same effects as gMDSC depletion. Similar gMDSC depletion and CTLA-4 blockade did not result in tumor control in non-T-cell inflamed MOC2 tumors, suggesting that rescuing or enhancing TIL function alone is likely not enough to induce anti-tumor immunity in TMEs that do not intrinsically support some degree of anti-tumor immunity at baseline. Supported by TCGA data analysis demonstrating a high MDSC gene expression profile in HNSCC patients with the most T-cell inflamed tumors, our data suggests that therapeutically targeting MDSCs in these tumors represents a rational strategy to enhance responses to CTLA-4-based checkpoint inhibition.

Several cell types may contribute to immunosuppression within the human HNSCC TME including tumor and various infiltrating immune cells [6]. Tregs are increased in the periphery and TME of patients with HNSCC, and mediate immunosuppression through defined mechanisms [8–10, 16]. Similarly, polarized (M2-like) macrophages that mediate immunosuppression through cytokine expression are increased in the TME of many solid tumor types including HNSCC [25, 26]. Clearly, cells other than MDSCs can contribute to immunosuppression within the HNSCC TME. However, infiltration of gMDSC inversely correlated with the presence of both Tregs and mature F4/80^+^ macrophages within the MOC1 TME, and gMDSC depletion completely rescued antigen-specific T-lymphocyte responses lost with tumor progression. There are Tregs present in the MOC TME very early in tumor progression when MDSC infiltration is relatively low, and we cannot rule out that these play an important role in establishing an immunosuppressive niche early in tumor development. Further, our finding that CTLA-4 mAb treatment reduced tumor infiltrating Tregs by roughly 50% suggests that Treg depletion may account for some of the immune stimulatory effects observed with CTLA-4 mAb treatment [19, 20]. Other studies have demonstrated that direct CD8^+^ T-lymphocyte activation is also required for CTLA-4 mAb efficacy [27]. Since most CD8^+^ TIL express CTLA-4 early in tumor progression and Treg levels are reduced after CTLA-4 mAb treatment, both direct CD8^+^ TIL activation and Treg depletion may be one advantage of CTLA-4 blockade over blockade of the PD-axis, as evidence suggests that PD inhibition primarily reverses adaptive immune resistance only [28, 29]. Ly6G depletion rescued antigen-specific T-lymphocyte responses and consistently sensitized T-cell inflamed MOC1 tumors to CTLA-4 mAb-induced rejection, suggesting that gMDSCs are the dominant cell type to contribute to immunosuppression associated with tumor progression within this model.

Efficient and specific depletion of Ly6G^hi^ gMDSCs from the spleen and TME was achieved with the 1A8 antibody up to 6 days following a single treatment. The RB6 antibody clone is known to be cross reactive with Ly6G and Ly6C [30], and many CD8 T-lymphocytes express cell surface Ly6C [31]. We demonstrated that systemic administration of RB6 mAb resulted in significant labelling of peripheral CD8^+^ T-lymphocytes. Others have demonstrated rapid rebound of MDSC populations following RB6 administration and the inability of RB6 to deplete MDSC from solid organs [32, 33]. These data suggest that administration of 1A8 is the method of choice for specific depletion of Ly6G^hi^ gMDSC from both the periphery and TME for mechanistic MDSC studies in mice.

Using an antibody-based cellular depletion approach to eliminate MDSCs from the human HNSCC TME is likely not feasible in patients. Several translational strategies to limit MDSC recruitment or function have been evaluated [reviewed in [34]]. Our data and the work of others [11, 12, 22] demonstrating that the CXCR2 signaling axis appears to play a significant role in the recruitment of gMDSCs in the TME suggests that disruption of this pathway has therapeutic potential. Expression of the CXCR2 ligands CXCL1 and CXCL2, known to be downstream of MAPK and NF-κB signaling [22, 35], correlated with accumulation of CXCR2 positive gMDSC in the TME with tumor progression. Analysis of the TCGA HNSCC dataset revealed that CXCR2, CSF1R and CTLA-4 are significantly expressed in HNSCC samples compared to other tumor types, and we validated the expression of CXCR2 on sorted peripheral blood gMDSC and CSF1R on mMDSC from an independent cohort of patients with advanced pharyngeal SCC. Subsets of patients with MDSC-rich gene expression profiles also demonstrated the highest expression of CD8^+^ T-lymphocyte associated genes. Thus, HNSCC patients with the highest MDSC tumor infiltration also harbor the most T-cell inflamed tumors, supporting the rational to limit MDSC recruitment or function to enhance responses to checkpoint inhibition in these patients. An MDSC-rich gene expression profile also correlated with higher CXCL2 and CSF1 expression, HPV-positivity, and advanced disease stage but not mutational burden. These findings also argue against a non-T-cell inflamed tumor status being due to increased recruitment of MDSC into the TME or a significantly lower genetic alteration rate, but rather point to a tumor cell intrinsic mechanism of immune escape in these tumors.

Limitations of our model include the lack of mMDSC overall, and the relative paucity of Tregs with tumor progression. This makes isolation and functional analysis of these cells types impractical. It is likely that the relative contribution of Tregs, mMDSC and M2-macrophages differs between pre-clinical models and that other syngeneic models would be more appropriate to study the relevance of these cell types. As a proof-of-principle, our work demonstrates that elimination of the dominant immunosuppressive cell type (gMDSCs) within a T-cell inflamed syngeneic system can sensitize tumors to CTLA-4 mAb induced rejection.

In conclusion, Ly6G^hi^ gMDSCs are a major driver of immunosuppression within the MOC TME that limit responses to CTLA-4-based checkpoint inhibition. Specific depletion of gMDSC rescued antigen-specific TIL responses lost with tumor progression. Consistent CD8^+^ T-lymphocyte-dependent rejection of established T-cell inflamed MOC1 but not non-T-cell inflamed MOC2 tumors was achieved with gMDSC depletion and CTLA-4 mAb and resulted in immunologic memory. This result appeared to be independent of PD-L1 despite high expression this checkpoint ligand on gMDSC. Correlatively, gMDSC recruitment into MOC1 tumors appeared to occur through CXCR2-axis signaling. These data, along with TCGA analyses demonstrating co-occurrence of an MDSC-rich gene expression profile and a T-cell inflamed tumor phenotype, strongly support combining MDSC-targeting therapeutic strategies with CTLA-4-based checkpoint inhibition in the clinical setting.

## MATERIALS AND METHODS

### *In vivo* tumor growth experiments

MOC cells were obtained from R. Uppaluri (Washington University in St. Louis) in 2014, have been validated to be of epithelial origin [36], were tested monthly for mycoplasma, cultured as described before [37] and used at low passage number ( < 30) for all experiments. To establish tumors, MOC1 (5×10^6^) or MOC2 (1×10^5^) cells were implanted subcutaneously (subq) in the flank. Our institute's Animal Care and Use Committee approved all experiments. *In vivo* Ly6G (clones RB6 and 1A8), CTLA-4 (clone 9H10), CD25 (clone PC-61.5.3), PD-L1 (clone 10F.9G2) or control (clone 2A3, BioXCell) mAbs were administered *via* intraperitoneal (IP) injection at 200 μg/injection (Ly6G, CD25 and PD-L1) or 100 μg/injection (CTLA-4). Challenge experiments were performed by injecting MOC1 cells (5×10^6^) subq in the contralateral flank. In some experiments, CD8 (clone YTS 169.4), CD4 (clone GK1.5) and NK (clone PK136) cells were depleted *via* IP injection (200μg/injection) twice weekly.

### Flow cytometry and tissue preparation

Spleens and lymph nodes were crushed between frosted slides and filtered (70 μM). Tumors were minced, digested using a mouse tumor dissociation kit (Miltenyi) per manufacturer protocol and filtered (40 μM). Following CD16/32 block (Biolegend), single cell suspensions were stained with primary antibodies. Fluorophore-conjugated primary antibodies included anti-mouse CD45.2 clone 104, CD11b clone M1/70, Ly-6C clone HK1.4, Ly-6G clone 1A8, Gr1 clone RB6-8C5, CD8 clone 53-6.7, NK1.1 clone PK136, CD4 clone GK1.5, FoxP3 clone FJK-16s, CD11c clone N418, F4/80 clone BM8, CD206 clone C068C2, CD19 clone 6D5, B220 clone RA3-6B2, CD107a clone 1D4B, CD25 clone PC61.5.3, PD-1 clone 29F.1A12, CTLA-4 clone UC10-4B9, TIM-3 clone RMT3-23, LAG-3 clone eBioC9B7w, CD27 clone LG.7F9, 4-1BB clone 17B5, ICOS clone C398.4A, OX-40 clone OX-86, CXCR2 clone SA044G4, CCR2 clone 475301, CSF1R clone AFS98, CD3 clone 145-2C11, PD-L1 clone 10F.9G2, CD44 clone IM7, CD69 clone H1.2F3, PDCA clone 129c1, I-A/I-E clone M5/114.152, H2-K^b^ clone AF6-88.5, and CD31 clone 390. Cells were stained with antibodies for one hour, washed, and analyzed by flow cytometry on a BD Canto using BD FACS Diva software. All cells stained for cell surface marker analysis were stained with 7AAD to determine viability, and isotype controls and a “fluorescence minus one” method were used to determine staining specificity. FoxP3^+^ regulatory CD4^+^ T-lymphocytes (T_reg_s) were stained using the Mouse Regulatory T Cell Staining Kit #1 (eBioscience) per manufacturer protocol. Staining for other intracellular targets was performed with the Fixation and Permeabilization Buffer Set (eBioscience) per manufacturer protocol. Post-acquisition analysis was performed with FlowJo vX10.0.7r2.

### Antigen-specific T-lymphocyte IFN production

T-lymphocytes were sorted using negative magnetic separation (Pan T Cell Isolation Kit II, Miltenyi) alone from tumor draining lymph nodes or following a 40/80% isotonic Percoll (Sigma) gradient from tumors. T-lymphocytes were consistently enriched from lymph node and tumor to ≥90% by flow cytometry. T-lymphocytes were added to IFNγ pretreated (24 hours, 20 ng/mL) and irradiated (50 Gy) parental tumor cells at a 10:1 E:T ratio and 48 hour supernatants were assessed for IFNγ concentration by ELISA (eBioscience) per manufacturer protocol. CD3/28 coated microbeads (Dynabeads, Thermo) at a 1:1 ratio for 24 hours were used as a positive control for T-lymphocyte stimulation.

### T-lymphocyte suppression assays

T-lymphocyte suppression assays were performed using plate-bound CD3 and CD28 antibodies as previously described [38]. gMDSC used for functional assays were harvested from mice 20-30 days after engraftment. To assess CTL function, OT-1 CTLs were generated by exposing OT-1 splenocytes to SIINFEKL peptide (2 μg/mL) with serial passaging for 72 hours. OT-1 CTLs were then combined with SIINFEKL pulsed and Indium^111^ labeled EL4 cells at a 10:1 E:T ratio, alone or in the presence of splenic or tumor infiltrating Ly6G+ cells, and CTL activity was quantified *via* indium release at 4 hours on a WIZARD2 Automatic Gamma Counter (PerkinElmer).

### Immunofluorescence

Frozen tumors embedded in OCT were sectioned to 5 μm and fixed for 15 minutes with ice-cold methanol. Following washings, sections nonspecific stained was blocked with a blocking solution (3% BSA + 0.1% Tween 20 in 1x PBS x 1 hour followed by 10% goat serum in 1x PBS for one hour). Sections were then incubated with a primary conjugated antibody diluted in blocking solution (1% BSA + 0.1% Tween 20 in 1x PBS) overnight at 4°C in a humidifying chamber. Primary antibodies included anti-CD8 (clone 53-6.7), CD11b (clone M1/70), Ly6G (clone A18) and CXCR2 (clone SA045E1). After washings, slides were mounted with Vectashield mounting medium with DAPI (Vector Laboratories) and analyzed on a LSM 780 confocal microscope (Carl Zeiss). Confocal images were analyzed using Zen 2012 SP1 software (black edition). Quantification of at least five high power fields was performed with ImageJ software.

### qRT-PCR

Whole tumor lysates were generated using the Tissue Lyser II and RNA was purified using the RNEasy Mini Kit (Qiagen, Valencia, CA) per the manufacturer's protocol. cDNA was synthesized utilizing a RT^2^ First Strand Kit (Qiagen). A customized RT^2^ PCR array (Qiagen) was used to assess the relative expression of target genes compared to GAPDH on a Viia7 qPCR analyzer (Applied Biosystems, Carlsbad, CA).

### TCGA analysis

Data from 279 HNSCC patients was extracted from the TCGA dataset. This included level 3 RNA-Seq data (presented as log2 transformed reads per kilobase of transcript per million mapped reads [RPKM]) and clinical data (HPV status, tumor stage, and tumor source site). Curated MAF files were downloaded from the Broad Institute Firebrowse website (http://firebrowse.org/). RNA-Seq data was subjected to hierarchical clustering based upon a supervised list of MDSC associated genes listed in alphabetical order [39]. Supervised analysis by Manhattan distance and Ward linkage method was performed using the Pheatmap package of the R software. On the displayed heatmap, the y-axis lists the MDSC-associated genes, with each column representing an individual patient. Red indicates relative overexpression of that gene for that patient relative to the mean expression of that gene for all patients, and blue represents relative underexpression. RNA expression patterns of effector CD8 T-lymphocyte associated genes (*CD8A, PRF1, GZMB, IFNG*) or chemokine receptor/ligands (*CXCR2, CSF1R, CXCL1, CXCL8, CSF1*) were compared among MDSC clustering subgroups (I-IV). Distributions of expression levels were analyzed for significance by 1-way ANOVA and plotted using GraphPad Prism v7. The non-synonymous (single nucleotide changes and frame shift indel mutations) mutational rate per Megabase (MB) was calculated based on accumulated counts of individual patient mutations divided by the total length of coding sequences. Distributions of mutation rates were analyzed for significance by 1-way ANOVA. Distributions of clinical parameters were analyzed for significance by χ-square analysis. RNA expression profiles of 22 tumor types were accessed through the FireBrowser (http://firebrowse.org/) web server and queried for *CTLA4, CXCR2, CSF1R*. Box and whisker plot of median expression distributions for each tumor type were presented as log2 RSEM (RNA-Seq by Expectation-Maximization).

### Analysis of sorted human MDSC

Monocytic and granulocytic MDSC were sorted from peripheral blood of patients with advanced HNSCC as described [14]. Brieftly, monocytic MDSC (CD11b^hi^, CD14^hi^, HLA-Dr^low^, CD15^neg^) and granulocytic MDSC (CD11b^hi^, CD14^neg^, HLA-DR^low^, CD15^hi^) populations from HNSCC were sorted from freshly obtained peripheral blood (PB), and/or tumor specimens from HNSCC patients undergoing surgical treatments or diagnostic procedures (such as biopsies) at Johns Hopkins Hospital and Johns Hopkins Bayview Hospital using MoFlo MLS sorter (Beckman Coulter) or a FACSAria II cell sorter (Beckton Dickinson). Samples were placed into Trizol (TRIzol^®^ Reagent), and RNA isolation was done using the RNeasy Mini Kit (QIAGEN, www.qiagen.com). These products were amplified and labeled with the Ovation Pico WTA Systems V2 kit and Encore Biotin Module as described in the manufacturer's manual (NuGEN Technologies Inc. San Carlos CA, USA). Affymetrix Human Gene 1.0 ST microarrays (Affymetrix, Santa Clara, CA) were hybridized for 16hrs at 45°C with rotation (60rpm) as described in Affymetrix’ GeneChip Expression Wash, Stain and Scan User Manual (www.affymetrix.com). Fluorescent signals were determined using Affymetrix’ GeneChip Scanner 3000 7G at default parameters described in the manufacturer's GeneChip Expression Analysis Technical Manual. Images were analyzed using the Affymetrix Command Console version 3 (AGCC v3.0), and processed into CEL files at the manufacturer's default settings. RMA normalized log2 signal values were extracted and summarized for gene-level analysis with the Partek Genomics Suite v6.6 (Partek Inc., St. Louis MO, USA). These gene expression data were compared between gMDSC and mMDSC by one-way ANOVA using Partek, and the results exported for examination and further evaluation to Spotfire DecisionSite (TIBCO Software Inc., Palo Alto CA, USA).

### Statistical analysis

Tests of significance between pairs of data are reported as p-values, derived using a student's *t*-test with a two-tailed distribution and calculated at 95% confidence. Comparison of multiple sets of data was achieved with analysis of variance with Tukey's multiple comparisons. Survival significance was determined by Log-Rank (Mantel-Cox) analysis. Error bars indicate standard error of measurement (SEM) for averaged tumor growth curves and standard deviation (SD) for all other data. All analysis was performed using GraphPad Prism v7.

## SUPPLEMENTARY MATERIALS FIGURES AND TABLES


