# Supplementary material for: Resistance to CTLA-4 checkpoint inhibition reversed through selective elimination of granulocytic myeloid cells
Source: Oncotarget. 2017 Jun 11;8(34):55804–20. doi: 10.18632/oncotarget.18437 (PMC5593525; doi:10.18632/oncotarget.18437)
Supplement: Supplementary file 1 [file oncotarget-08-55804-s001.pdf]

Resistance to CTLA-4 checkpoint inhibition reversed through selective elimination of granulocytic myeloid cells

SUPPLEMENTARY MATERIALS

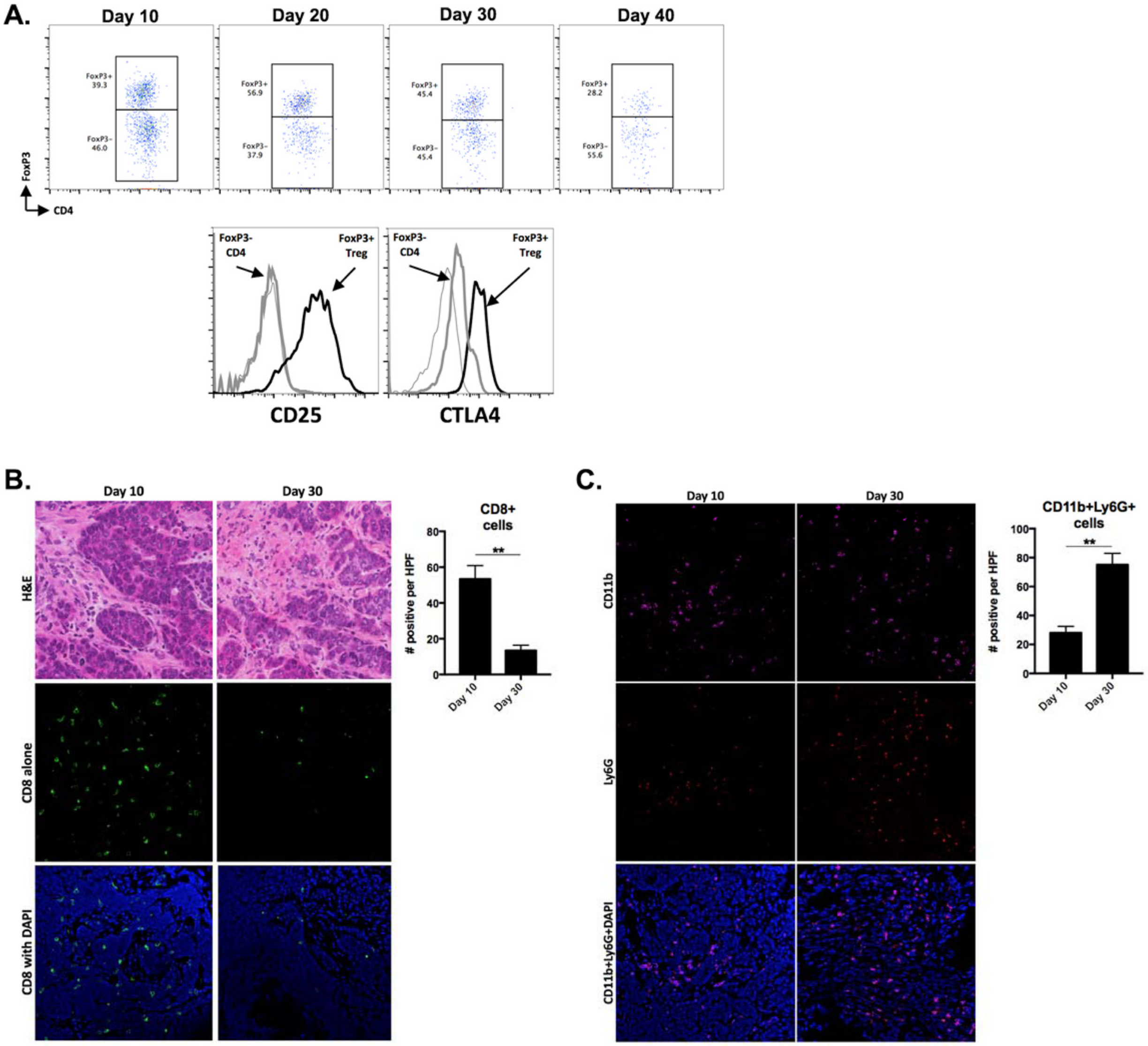

Supplementary Figure 1: Tumor accumulation of CD25 and CTLA-4 positive FoxP3+CD4+ Tregs decreased with tumor progression and infiltration of CD8+ T-lymphocytes and Ly6G+ myeloid cells by immunofluorescence correlated with flow cytometry findings. A, Representative dotplots of CD4$^+$ TIL from tumors harvested 10, 20, 30 or 40 days after tumor implantation. Representative histograms of CD25 and CTLA4 expression on FoxP3 positive and negative CD4$^+$ TIL. Thin grey line is isotype control. Tumors from day 10 and 30 after tumor implantation were sectioned and analyzed for CD8$^+$ (B) and CD11b$^+$Ly6G$^+$ (C) cell infiltration by immunofluorescence. Top left panels are H&E stained. Bottom panels counterstained with DAPI. Images obtained at 20x on a confocal microscope. Quantified with ImageJ software.

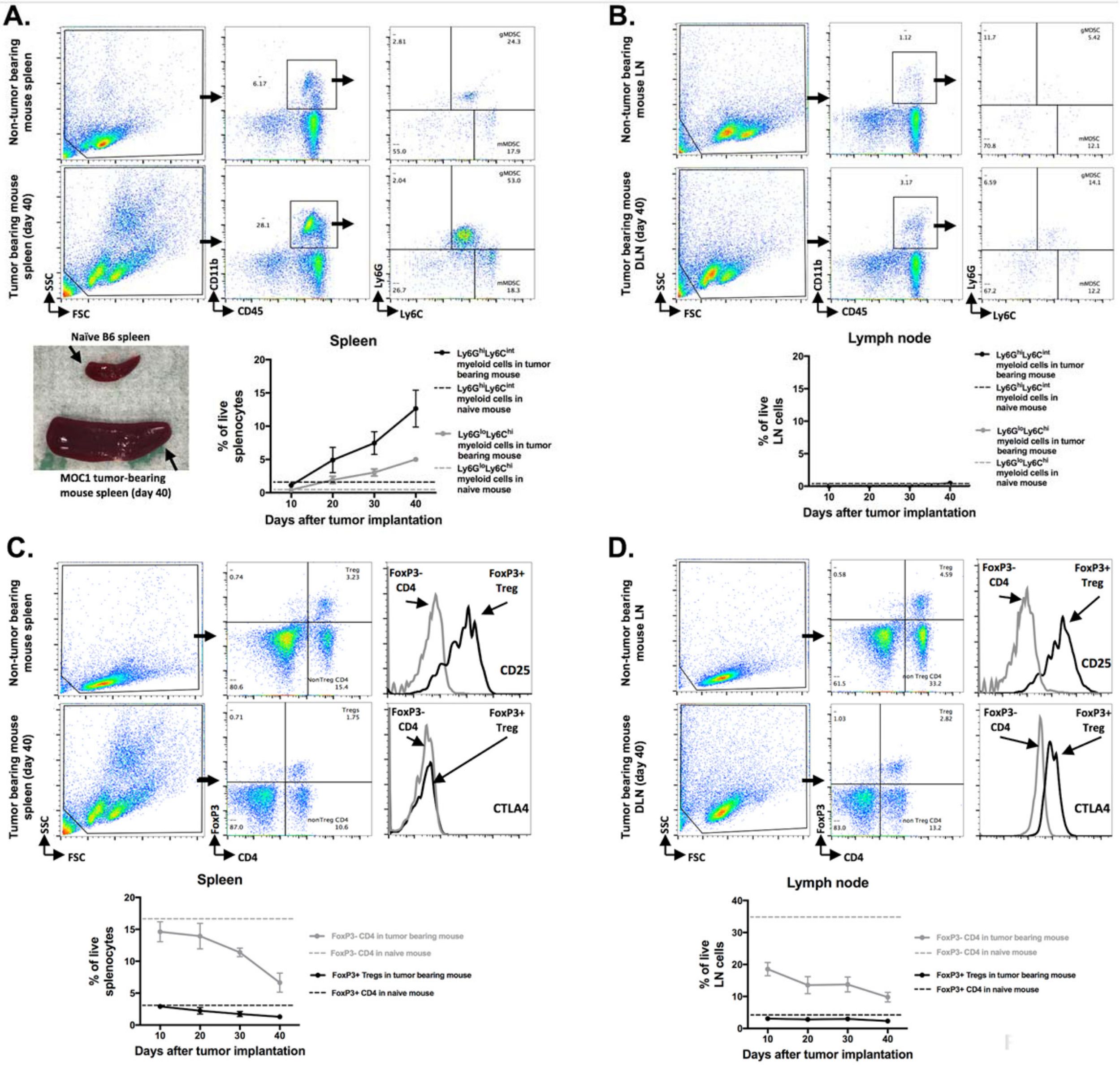

Supplemental Figure 2: Spleens of MOC1 tumor-bearing mice accumulated Ly6Ghi myeloid cells but not Tregs with tumor progression. Gating strategy (dotplots) and quantification of Ly6G^{hi}Ly6C^{int} myeloid cells and Ly6G^{lo}Ly6C^{hi} myeloid cells in spleens (A) and tumor-draining lymph nodes (B) of MOC1 tumor-bearing mice at days 10, 20, 40 and 40 after tumor implantation (n=5/ time point). Dotted lines represent levels in naïve non-tumor bearing mice. Photograph of a representative naïve and MOC1 tumor-bearing mouse spleen. Gating strategy (dotplots) and quantification of T$_{reg}$s in spleens (C) and tumor-draining lymph nodes (D) of MOC1 tumor-bearing mice. Representative histograms of CD25 and CTLA4 expression on FoxP3 positive and negative CD4^{+} T-lymphocytes.

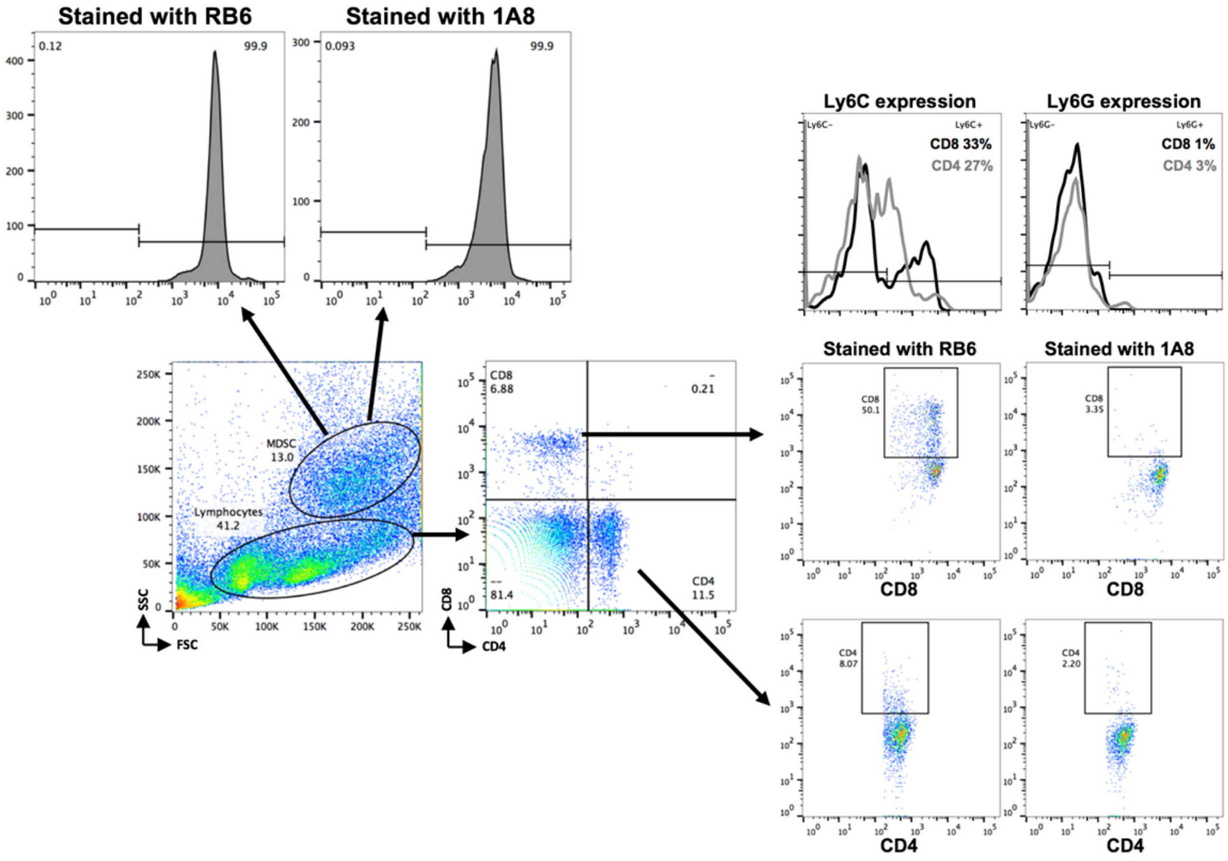

Supplementary Figure 3: The 1A8 but not RB6-8C5 antibody selectively bound to Ly6G. Spleens from a MOC1 tumor-bearing mouse (day 40) were stained with both 1A8 and RB6-8C5 antibodies. Both 1A8 and RB6-8C5 antibodies bind MDSCs gated from the FSC/SSC plot (top left panels). $CD4^+$ and $CD8^+$ lymphocytes were similarly stained (right bottom panels) with 50% of $CD8^+$ and 8% of $CD4^+$ T-lymphocytes also staining positive with the RB6 but not the 1A8 antibody. A significant subset of $CD8^+$ and $CD4^+$ T-lymphocytes express Ly6C but do not express Ly6G (right top panels).

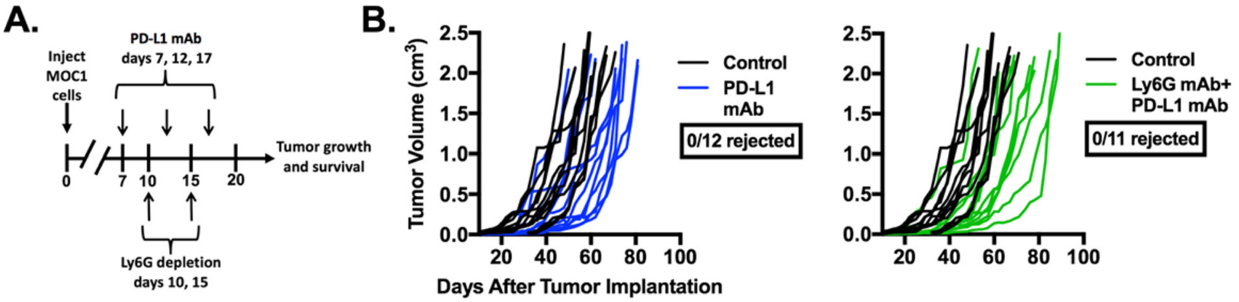

Supplementary Figure 4: gMDSC depletion plus PD-L1 mAb does not effectively control established MOC1 tumors. Established MOC1 tumors were treated with Ly6G depleting antibody (clone 1A8, 200 µg/injection) and PD-L1 mAb (clone 10F.9G2, 200 µg/injection), alone or in combination . A, schematic of Ly6G depletion and checkpoint blockade. B, primary tumor growth plots demonstrate growth curves for treated MOC1 tumors (colored lines) compared to control (black lines).
